# Data and model considerations for estimating time-varying functional connectivity in fMRI

**DOI:** 10.1016/j.neuroimage.2022.119026

**Published:** 2022-05-15

**Authors:** C Ahrends, A Stevner, U Pervaiz, ML Kringelbach, P Vuust, MW Woolrich, D Vidaurre

**Affiliations:** a Department of Clinical Medicine, Center for Music in the Brain, Aarhus University & Royal Academy of Music Aarhus/Aalborg, Universitetsbyen 3, Aarhus C 8000, Denmark; b Department of Clinical Medicine, Center of Functionally Integrative Neuroscience, Aarhus University, Universitetsbyen 3, Aarhus C 8000, Denmark; c Nuffield Department of Clinical Neurosciences, Oxford Centre for Functional MRI of the Brain (FMRIB), Wellcome Centre for Integrative Neuroimaging, University of Oxford, John Radcliffe Hospital, Headington, Oxford OX3 9DU, United Kingdom; d Department of Psychiatry, University of Oxford, Warneford Hospital, Warneford Ln, Headington, Oxford OX3 7JX, United Kingdom; e Department of Psychiatry, Oxford Centre for Human Brain Activity (OHBA), Wellcome Centre for Integrative Neuroimaging, University of Oxford, Warneford Hospital, Warneford Ln, Headington, Oxford OX3 7JX, United Kingdom

**Keywords:** fMRI, Time-varying FC, Hidden Markov Model (HMM), Resting state

## Abstract

Functional connectivity (FC) in the brain has been shown to exhibit subtle but reliable modulations within a session. One way of estimating time-varying FC is by using state-based models that describe fMRI time series as temporal sequences of states, each with an associated, characteristic pattern of FC. However, the estimation of these models from data sometimes fails to capture changes in a meaningful way, such that the model estimation assigns entire sessions (or the largest part of them) to a single state, therefore failing to capture within-session state modulations effectively; we refer to this phenomenon as the model becoming static, or model stasis. Here, we aim to quantify how the nature of the data and the choice of model parameters affect the model's ability to detect temporal changes in FC using both simulated fMRI time courses and resting state fMRI data. We show that large between-subject FC differences can overwhelm subtler within-session modulations, causing the model to become static. Further, the choice of parcellation can also affect the model's ability to detect temporal changes. We finally show that the model often becomes static when the number of free parameters per state that need to be estimated is high and the number of observations available for this estimation is low in comparison. Based on these findings, we derive a set of practical recommendations for time-varying FC studies, in terms of preprocessing, parcellation and complexity of the model.

## Highlights

• Time-varying FC models sometimes fail to detect temporal changes in fMRI data.
• Between-subject and within-session FC variability affect model stasis.
• The choice of parcellation affects model stasis in real fMRI data.
• The number of observations and free parameters per state critically affect model stasis.

## 1 Introduction

Neural circuits across multiple brain areas integrate into large-scale brain networks in order to accomplish complex cognitive functions. Just like smaller populations of neurons underlying these networks flexibly synchronise and desynchronise their oscillatory firing patters to communicate (Fries, 2005), large-scale brain networks must also be able to fluctuate dynamically and change over time (Breakspear, 2017; Calhoun et al., 2014), enabling flexible neuronal communication and functioning across the entire brain. Arguably, this is reflected in the data as some form of synchrony in the activity across areas, which is typically referred to as functional connectivity (FC). In fMRI, FC can be derived by measuring how different areas coactivate in their blood oxygen level dependent (BOLD) signal. Understanding these temporal changes in FC (i.e. time-varying FC) in fMRI can help to address a range of questions, from the theoretical study of human cognition to a better characterisation of different neurological and psychiatric diseases.

There are several approaches to modeling time-varying FC in fMRI; for a recent review, see Lurie et al. (2019). One avenue is the use of state-based models that estimate time-varying FC as a temporal sequence of brain “states”. These models reduce complex brain dynamics into a subset of patterns of transitions between quasi-stationary states, which can be statistically described and compared between groups of subjects or used to study conditions in a relatively straightforward way. While approaches using a single, more complex model to describe the entire time series (for instance a convolutional neural network or a high-order multivariate autoregressive model) can explain the data equally well or even better in terms of explained variance, a state-based model gives an advantage in interpretation if one is interested in disambiguating signal changes in a time-resolved manner (for example what happens and when as a response to a stimulus). However, in fMRI, these models are not always effective to detect changes in FC over time. Sometimes, the estimation leads to entire sessions collapsing into one single state, with no changes within session —so that the model becomes static; that is, all the explanatory power of the model is focussed on explaining differences between subjects or sessions, instead of within-session modulations. The reason behind this behavior is an open question: it could be because there are no temporal changes in FC in the data; or it could be because, even if there are temporal changes, the estimation is unable to detect them. While some studies have claimed that there is insufficient evidence that BOLD FC is dynamic (Hindriks et al., 2016; Liégeois et al., 2017; Lindquist et al., 2014), several studies have shown that dynamic aspects of FC are relevant for behavior and cognition (Cabral et al., 2017; Fornito and Bullmore, 2010; Gonzalez-Castillo and Bandettini, 2018; Karapanagiotidis et al., 2020; Liegeois et al., 2019; Vidaurre et al., 2017; Voytek and Knight, 2015; Xie et al., 2018) and that they can add important information not contained in time-averaged FC (Vidaurre et al., 2021). These findings suggest that temporal variation is present in BOLD FC and that it carries meaningful information. We here address the question of how to quantify this variability effectively, and under which conditions the models become static (i.e. when “model stasis” occurs), since a deeper understanding of the issue can help us to configure these models to work more optimally.

**Fig. 1:**
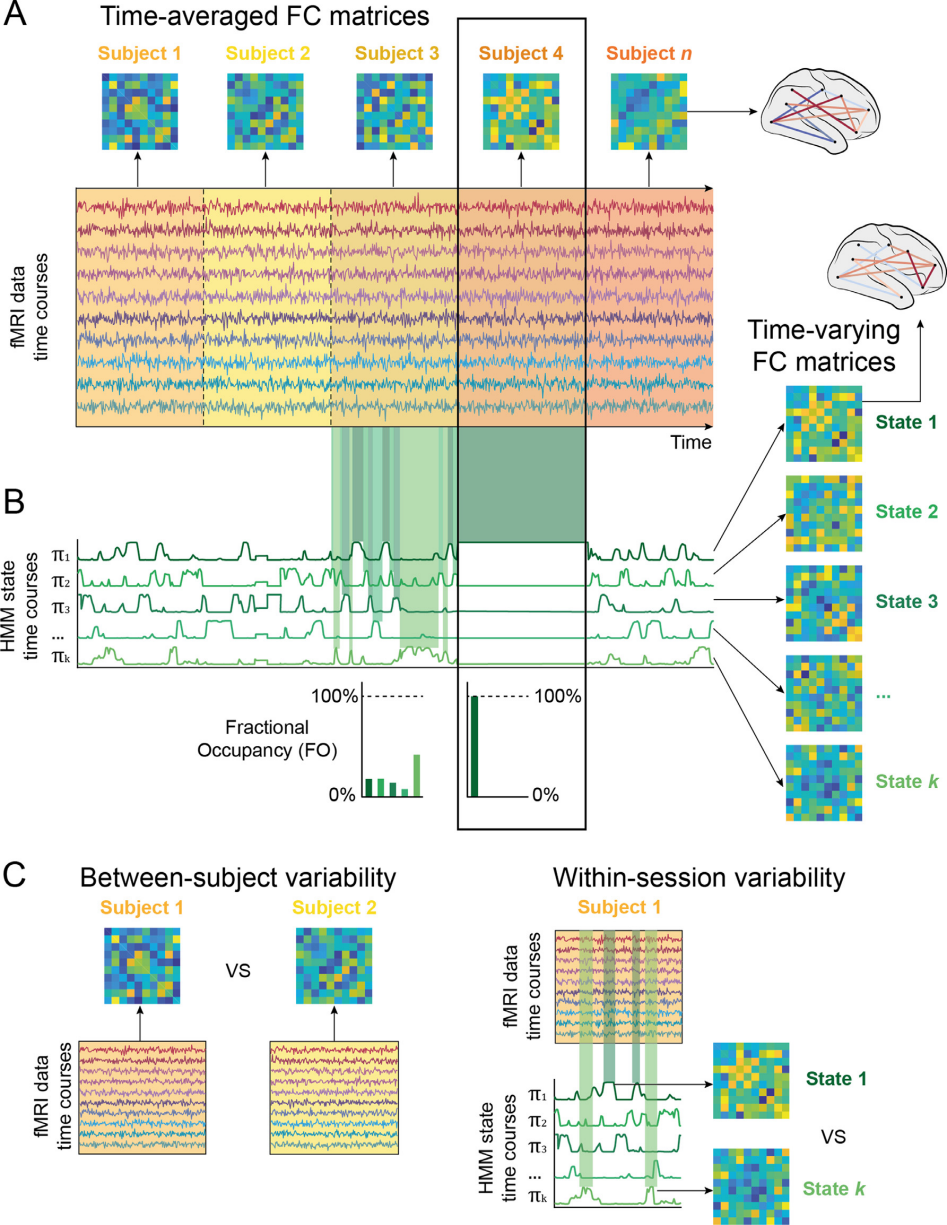
Between-subject variability in time-averaged FC may affect stasis in a time-varying FC model. (A) Time-averaged FC (N by N) matrices for each subject were obtained by pairwise correlating time courses of all parcels from each subject. Subjects are represented in the time series as different colors. As observed, the time-averaged FC matrix from Subject 4 is very different from the time-averaged FC matrices of the other subjects. (B) Given a prespecified number of states K, the Hidden Markov Model (HMM) estimates both the state-specific FC matrices and when the states become active. In the example for Subject 3, all states transiently occur and recur over time. In opposition to this temporal recurrence in Subject 3, the HMM time course for the time points corresponding to Subject 4 stays stable at a high probability for state 1. The temporal recurrence of states can be measured by their fractional occupancy (FO), indicating the proportion of the entire time series that a given state occupies. In this example, state FOs for Subject 3 indicate that all states take up a similar amount of the time series with certain states being relatively more prevalent than others. In Subject 4, however, the FO of state 1 is at 100% while all others are at 0%, since state 1 occupies the entire time series of this subject. This is summarised by the term “stasis”: The model is static when one state's FO approaches 100% and all others are close to 0%. (C) Between-subject variability refers to differences in time-averaged FC between subjects. For instance, we can compute the FC over all timepoints of Subject 1 and compare it to the FC of all timepoints from Subject 2. Within-session variability refers to differences in FC between timepoints in a session. For instance, the HMM identifies timepoints, at which State 1 is active, and timepoints, at which State k is active, in Subject 1′s session. We can then compare the FC of State 1 with the FC of State k.

Assuming temporal FC changes exist in the data, why would a time-varying FC model fail to detect them? One possible explanation is in the nature of the data. We here refer to factors that affect the data as the **data hypothesis**. In particular, since unsupervised, data-driven time-varying FC models aim at describing the most salient patterns in the data, within-session fluctuations might just be too subtle, with overall differences between subjects being more dominant (Lehmann et al., 2017). That is, if *between*-subject differences are larger than *within*-session FC modulations (i.e. changes over time within a subject's scanning session), a data-driven model will naturally prefer to focus on the between-subject variability instead of the temporal variability. By between-subject variability, we here mean time-averaged FC differences between subjects. By within-session variability, we here mean temporal fluctuations in FC, i.e. differences between timepoints within each subject's scanning session. This is illustrated in Fig. 1C. As we will show, the balance between these two aspects of variability (between-subject and within-session) depends on the preprocessing pipeline, in particular on the choice of a parcellation (Eickhoff et al., 2018; Pervaiz et al., 2020; Popovych et al., 2021), and how fine-grained it is. Another explanation relates to challenges in estimating the model; i.e. if the model inference has problems in finding within-session modulations. We refer to this explanation as the **estimation hypothesis**, which, in particular, might occur when the number of free parameters per state to estimate in the model is too large in comparison to the available number of volumes or time points (across subjects).

In the present study, we simulated data with varying amounts of variability between and within subjects, and we fitted models to a real dataset in different parcellations. We hypothesise that large between-subject variability and small within-session (temporal) variability cause the time-varying FC model to become static and that this effect depends on the parcellation (data hypothesis). We further hypothesise that fewer observations and more free parameters per state, in fact a small ratio of number of observations to free parameters per state, cause the time-varying FC model to become static (estimation hypothesis). We finally provide some recommendations for the estimation of time-varying FC based on these points.

## 2 Material and methods

### 2.1 Data and parameters

#### 2.1.1 HCP dataset and preprocessing

We used resting state EPI scans of the first 200 participants from the Human Connectome Project S1200 (HCP, Smith et al., 2013b; Van Essen et al., 2013), an open-access dataset of MRI data. Time-varying FC has previously been demonstrated in this dataset using a wide array of different approaches (Battaglia et al., 2020; Casorso et al., 2019; Choe et al., 2017; Dai et al., 2019; Liegeois et al., 2019; Riccelli et al., 2017; Sporns et al., 2021; Vidaurre et al., 2017; Zalesky et al., 2014; Zamani Esfahlani et al., 2020), making it a suitable example to evaluate model stasis. The dataset consists of structural and functional MRI data of 1200 healthy, young adults (age 22–35). Each participant completed four resting state scans. We here only used data from the first resting state scanning session of each participant. Data were acquired as described in the HCP public protocols, which can be found in Van Essen et al. (2012). Briefly, scans were acquired in a 3T MRI scanner, using multiband echo planar imaging sequences with an acceleration factor of 8 at 0.72 s repetition time (TR) and a spatial resolution of 2 × 2 × 2 mm for functional scans. Resting state scans lasted 14 min and 33 s.

Data were preprocessed following the HCP preprocessing pipelines for resting-state fMRI (Glasser et al., 2013; Smith et al., 2013a). In brief, after “minimal” spatial preprocessing and surface projection to transform data into grayordinate space, the data were temporally preprocessed using single-session Independent Component Analysis (ICA, using FSL's MELODIC; Beckmann, 2012), as well as classification and removal of noise components using FSL's FIX (Griffanti et al., 2014; Salimi-Khorshidi et al., 2014).

#### 2.1.2 Parcellations and time course extraction

Group ICA parcellations estimate a data-driven functional parcellation on the group level, which are subsequently regressed onto each subject's individual functional scans to obtain subject-specific versions of group ICs and their time courses. Group ICA parcellations were created for a varying number of parcels (we here used the variants created for 50 and 100 parcels, GroupICA50 and GroupICA100) using multi-session spatial ICA on the temporally concatenated data. The time series for each participant were extracted using dual regression (Beckmann et al., 2009). The Group ICA parcellations and corresponding time series are publicly available from the HCP repository (https://db.humanconnectome.org).

PROFUMO (Harrison et al., 2015) is a similar approach to Group ICA, but it estimates group- as well as subject-level maps simultaneously, allowing it to better capture individual variability in FC (Bijsterbosch et al., 2018). In PROFUMO, between-subject differences in (time-averaged) FC are therefore expected to be higher compared to the group ICA approach. We used a PROFUMO parcellation of 50 parcels, PROFUMO50.

As *a priori* defined functional parcellation, we used the Yeo parcellation (Schaefer et al., 2018). This parcellation was created using a gradient-weighted Markov Random Field on a separate dataset of resting-state fMRI recordings of 1489 participants. This approach produces parcels which are similar in terms of function and connectivity. We here used the grayordinate version of this parcellation consisting of 100 parcels (Yeo100 parcellation).

As an anatomical parcellation, we used the Desikan-Killiany atlas (Desikan et al., 2006). This atlas originally consists of 62 anatomically delineated cortical regions. The atlas was projected into grayordinate space and 18 subcortical regions were added, as described in Deco et al. (2021). This resulted in 80 parcels (DK80 parcellation). Time courses in this parcellation were extracted as the mean across grayordinates belonging to each parcel.

Beside runs that use the full parcellations, we also ran the models on subsets of each parcellation to vary the number of free parameters per state in the model (as described under Section 2.3). In these reduced runs, we randomly chose a subset of 10, 25, or 50 parcels from a parcellation, which time series were subsequently fed to the model. This random selection was repeated five times for each subset of a parcellation. As an alternative strategy to reduce the number of free parameters per state in the model, we also tested the effects of reducing the original data dimensionality using Principal Component Analysis (PCA) or by modeling each HMM-state as probabilistic PCA model (“HMM-PCA”) (Vidaurre, 2021).

To mimic properties of more ordinary datasets, we also varied the number of subjects (S) between 50, 100, and 200, the number of time points (T) per subject between 200, 500, and 1200 time points, and the fraction R of the sampling rate at 1.37 Hz (original rate R=1, equivalent to TR of 0.72 s), 0.68 Hz (half of the original rate $R=\frac{1}{2}$, equivalent to TR of 1.44 s), and 0.46 Hz (one third of the original rate $R=\frac{1}{3}$, equivalent to TR of 2.16 s). The number of observations O used in the model is the total amount of time points: O=S*T*R. In our analysis, we modeled only the effect of the number of observations O, rather than the effects of the number of subjects, of time points, and of the sampling rate separately.

Time course extraction results in one matrix of dimensions T x N per subject, where N is the number of parcels. To compute time-varying FC, we concatenated the time series across subjects, resulting in a matrix of (S x T) x N. The input and size at this step varies with the parcellation, i.e. N=50 for the GroupICA50 and PROFUMO50 parcellations, N=80 for the anatomical DK80 parcellation, and N=100 for the GroupICA100 and Yeo100 parcellations in the full runs (i.e. with all regions or components). In the reduced runs, N corresponds to the number of randomly chosen parcels from each parcellation (10, 25, or 50 parcels). We then standardised these time series row-wise by rescaling them so that the time course of each parcel has a mean of 0 and a standard deviation of 1.

#### 2.1.3 Simulations

To be able to test the different levels of between-subject and within-session variability, we simulated new datasets based on the HCP data, where we introduced differing amounts of between-subject and within-session variability into the generative model. This was done by generating new time series from a combination of synthetic covariance matrices, representing either time-invariant (subject-specific) FC matrices or time-varying FC matrices that activate or deactivate at different time points:

$$X=0.5Y_{\mathrm{bs}}+0.5Z_{\mathrm{ws}}\;X,Y_{\mathrm{bs}},Z_{\mathrm{ws}}\in R^{\mathrm{Nx}\left(\mathrm{SxT}\right)}$$

Here, X is the synthetic time series containing variability both between subjects and within sessions, $Y_{bs}$ is the synthetic time series containing only variability between subjects, and $Z_{ws}$ is the synthetic time series containing only variability within sessions. In this notation, X, $Y_{bs}$, and $Z_{ws}$ all represent subjects’ individual time series that have been concatenated.

The time series $Y_{bs}$, containing only variability between subjects, was generated by randomly sampling from a Gaussian distribution with mean 0 and a different synthetic covariance matrix per subject:

$$Y_{bs}^{s}\;∼N\left(0,{\hat{\Sigma }}_{bs}^{s}\right)\;Y_{bs}^{s}\in R^{NxT},\;{\hat{\Sigma }}_{bs}^{s}\in R^{NxN}$$

where $Y_{bs}^{s}$ is the time series for subject s, and ${\hat{\Sigma }}_{bs}^{s}$ is the (symmetric, positive-definite) covariance matrix of subject s, i.e. containing FC information specific to this subject and different from the others.

The time series $Z_{ws}$, with only variability within a session, was obtained by sampling from an HMM distribution:

$$Z_{ws}^{s}\;∼HMM\left(\Theta \right)\;Z_{ws}^{s}\in R^{NxT}$$

where $Z_{ws}^{s}$ is the time series for subject s. Critically, $Z_{ws}^{s}$ contains only within-session variability, since the HMM parameters Θ are at the group level (i.e. equal for all subjects). More specifically, when a given state k is active, $Z_{ws}^{s}$ is sampled from a Gaussian distribution with mean 0 and a state-specific synthetic covariance ${\hat{\Sigma }}_{ws}^{k}$:

$$z_{t}^{s}|q_{t}^{s}=k\;∼N\left(0,{\hat{\Sigma }}_{ws}^{k}\right)\;z_{t}^{s}\in R^{Nx1},\;q_{t}^{s},k\in R\;\left\{1,\ldots K\right\},\;{\hat{\Sigma }}_{ws}^{k}\in R^{NxN}$$

Therefore, ${\hat{\Sigma }}_{ws}^{k}$ accounts for state-specific variability, which depends on the currently active state $q_{t}^{s}$. The currently active state $q_{t}^{s}$ depends on which state was active at the previous time point $q_{t-1}^{s}$. The states are sampled from a categorical distribution with the parameters A, which are the transition probabilities of the HMM ($A_{k}$ indicating the k-th row of the transition probability matrix):

$$q_{t}^{s}|q_{t-1}^{s}=k∼Cat\left(A_{k}\right)\;q_{t-1}^{s}\in R\;\left\{1,\ldots K\right\},A\in R^{KxK}$$

To create the covariance matrices ${\hat{\Sigma }}_{bs}^{s}$ and ${\hat{\Sigma }}_{ws}^{k}$, we first decomposed the real covariance matrix of the first subject of the HCP dataset into its singular values:

$$\Sigma =\mathrm{UD}U^{\prime }\;\Sigma ,U,D\in R^{\mathrm{NxN}}$$

where Σ is the covariance matrix of the first subject of the real dataset (HCP resting-state fMRI dataset) in GroupICA50 parcellation, U are the singular vectors of the covariance matrix and D contains the singular values of the covariance matrix.

We created synthetic covariance matrices ${\hat{\Sigma }}_{bs}^{s}$ for all subjects S by multiplying the original singular values D with subject-specific singular vectors ${\hat{U}}_{bs}^{s}$, which we created by randomly perturbing U.

$${\hat{\Sigma }}_{\mathrm{bs}}^{s}={\hat{U}}_{\mathrm{bs}}^{s}D{\hat{U}}_{\mathrm{bs}}^{s\prime }\;{\hat{U}}_{\mathrm{bs}}^{s}\in R^{\mathrm{NxN}}$$

Similarly, to create the covariance matrices ${\hat{\Sigma }}_{ws}^{k}$ for all states K, we multiplied the original singular values D with state-specific singular vectors ${\hat{U}}_{ws}^{k}$:

$${\hat{\Sigma }}_{\mathrm{ws}}^{k}={\hat{U}}_{\mathrm{ws}}^{k}D{\hat{U}}_{\mathrm{ws}}^{k\prime }\;{\hat{U}}_{\mathrm{ws}}^{k}\in R^{\mathrm{NxN}}$$

For each subject s, the noisy singular vectors ${\hat{U}}_{bs}^{s}$ were generated by multiplying the original singular vectors U element-wise with a subject-specific Gaussian noise matrix $\Psi^{s}$ and adding this product to the original vectors U:

$${\hat{U}}_{\mathrm{bs}}^{s}=U+U\circ \delta_{\mathrm{bs}}\Psi^{s}]\;\Psi^{s}\in R^{\mathrm{NxN}},\delta_{\mathrm{bs}}\in R\;\left[0.1,1\right]$$

The Gaussian noise matrix $\Psi^{s}$ is scaled by the parameter $\delta_{bs}$, which defines the final amount of between-subject variability contained in the synthetic time series $Y_{bs}$.

Similarly, for each state k, we generated the noisy singular vectors ${\hat{U}}_{ws}^{k}$ by multiplying the original singular vector U element-wise with a state-specific Gaussian noise matrix $\Psi^{k}$:

$${\hat{U}}_{\mathrm{ws}}^{k}=U+U\circ \delta_{\mathrm{ws}}\Psi^{k}\;\Psi^{k}\in R^{\mathrm{NxN}},\;\delta_{\mathrm{ws}}\in R\;\left[0.1,1\right]$$

This Gaussian noise matrix $\Psi^{k}$ is scaled by the parameter $\delta_{ws}$, which defines the amount of within-session variability contained in the synthetic time series $Z_{ws}$.

We varied the parameters $\delta_{bs}$ and $\delta_{ws}$ between 0.1 and 1 in steps of 0.1. A small value for $\delta_{bs}$ results in a time series $Y_{bs}$, in which all subjects’ time-averaged FC matrices are similar. A large value for $\delta_{bs}$, on the other hand, results in a time series $Y_{bs}$, in which subjects’ FC matrices are very different from each other. A small value for $\delta_{ws}$ results in a time series $Z_{ws}$, in which FC almost does not vary over time (i.e. FC is essentially static). A large value for $\delta_{ws}$, on the other hand, results in a time series $Z_{ws}$, in which FC varies greatly over time. Although we use a specific, real subject's covariance matrix as the basis for the simulations, the concrete FC configuration does not affect our analyses, since we only consider the relative effect of introduced between-subject and within-session variability.

We generated time series from all combinations X of $Y_{bs}$ and $Z_{ws}$, resulting in 100 simulated time series. We then used these time series as input to compute time-averaged FC, as described under Section 2.2, and to the time-varying FC model to evaluate the model's stasis, as described under Section 2.3.

### 2.2 Time-averaged functional connectivity and FC similarity

To compute time-averaged functional connectivity, Pearson's correlation was computed for each pair of regions (Smith et al., 2013c). The resulting N x N matrices represent the time-averaged FC of each scanning session within each parcellation. In order to assess how consistent these FC networks were for each of the parcellations, we estimated the network similarity across scanning sessions. This was done by first calculating the group average of the time-averaged FC in each parcellation, then unwrapping the upper triangular elements of this group average FC matrix into a $\left[\frac{N^{2}-N}{2}\right]$ x 1 vector, and correlating this group-level vector with the corresponding vectors of the session-specific FC matrices. For each parcellation, FC similarity was thus defined as the correlation between the group mean FC, and the FC of all individual scanning sessions.

### 2.3 Time-varying functional connectivity: Hidden Markov Model (HMM) and model stasis

We used the Hidden Markov Model (HMM; Vidaurre et al., 2016, 2017) to describe time-varying FC. The HMM is a type of state-based model that estimates a sequence of states and a probability distribution for each state, such that each time point in the time series is assumed to have been generated from its assigned state distribution. The HMM has been used to estimate time-varying FC on fMRI and MEG data in previous work (Karapanagiotidis et al., 2020; Quinn et al., 2018; Stevner et al., 2019; Vidaurre et al., 2016, 2017).

We used a version of the HMM that assumes a multivariate Gaussian distribution per state, with K=6 states for the simulated data and K=12 for the HCP data. We additionally investigated the effect of the number of states K on model stasis by varying K∈R{4,…60} in steps of 4. In order to focus on FC, each state was here defined in terms of its covariance only (Vidaurre, 2021), i.e. by setting the mean of each state to zero and not allowing the inference to change this value. Once the model was estimated, we computed the fractional occupancy (FO), defined as the proportion that each state occupies in the time series of a particular subject. We used FO as indicator of the model becoming static. This is illustrated in Fig. 1. In the example, different states are assigned to portions of the time series of Subject 3, resulting in a small FO percentage for each of the states. For Subject 4, however, a single state (#1) is assigned to all time points of this subject, i.e. the FO of state #1 in Subject 4 is 100%. This means that the model effectively fails in finding any temporal changes in functional connectivity for this subject, describing only the more salient difference between all time points of this subject compared to the other subjects. To evaluate the model's overall stasis, we then used the maximum FO value of each subject and computed the average across the group. In practice, this means that a model that assigns states only to entire subjects, such as in the example with Subject 4, will have a mean maxFO of 100%. On the other hand, a model that finds recurring states over time that are perfectly equally distributed across time points of all subjects will have a mean maxFO of approximately $\frac{1}{K}$ (i.e. each of the K states occupies on average the same amount of each subject's time series). We here used stasis, as measured by the model's mean maxFO, as an indicator of how well a time-varying FC model is able to estimate temporally recurring states. While the mean maxFO across the group does not capture the full distribution of maxFOs in all subjects, we use it as a summary measure to be able to represent model stasis of each model as a single value. Example distributions of maxFO values across groups of subjects and the corresponding mean maxFO are shown in Supplementary Fig. 3.

To test our estimation hypothesis, we calculated the number of free parameters per state of each model. If this number is too large in comparison to the number of observations O, the estimation may become statistically challenging. The number of free parameters per state DF in a HMM with K states, each defined by a full covariance matrix but without modeling the mean, and N parcels can be computed as

$$DF=\frac{K*\left(K-1\right)+\left(K-1\right)+\frac{K*N*\left(N+1\right)}{2}}{K}.$$

We implemented the model using the HMM-MAR toolbox available at https://github.com/OHBA-analysis/HMM-MAR in MATLAB (The Mathworks Inc, 2016). Although the HMM is only one example of a time-varying FC model, the concepts discussed here are likely to apply also to other models of time-varying FC.

### 2.4 Structural equation modeling (SEM)

To provide a synthesis of the hypothesised relationships, we modeled all effects in a structural equation model (SEM). SEM characterises the causal links between variables, which are combined in a network of structural equations. In these structural equations, the relationships between variables are explicitly declared. Each variable can be declared to have a direct effect on an outcome variable or an indirect effect by declaring this variable simultaneously as an outcome and a predictor variable. A single variable can also have both a direct and an indirect effect on an outcome variable. Here, we combined a series of linear models and linear mixed effects models in a piecewise SEM, also called confirmatory path analysis (Shipley, 2000). Rather than estimating coefficients in a single variance-covariance matrix as in traditional SEM, piecewise SEM first estimates each part of the model independently before evaluating them at the level of the full model. This allows increased flexibility on the level of the constituting parts of the SEM in terms of their distributions, making it possible e.g. to include random effects in parts of the model.

We fitted two separate SEMs: one to the outcomes of HMMs run on simulated data and one to the outcomes of HMMs run on the real (HCP) data. The structures of the SEMs are illustrated in Fig. 5. In both SEMs, there are two parts. The first part constitutes the effect of the observed variables on FC similarity, and the second part links the observed variables and FC similarity to mean maxFO as an indicator of the HMM's model stasis. In the SEM on simulated data, the first part modeled the effects on FC similarity of two factors: 1. the number of observations O (which here depends only on the number of subjects S) and 2. between-subject variability (the value of the parameter $\delta_{bs}$, described under Section 2.1.3). The second part modeled the effects on model stasis of four factors: 1. the number of observations O, 2. FC similarity, 3. within-session variability (the value of the parameter $\delta_{ws}$, described under Section 2.1.3), and 4. the inverse of the number of free parameters per state DF (which varies based on the number of parcels N from each parcellation). The number of observations O has therefore both a direct effect on model stasis and an indirect effect via FC similarity. In the SEM on real data, the first part modeled the effect on FC similarity of only one factor: the number of observations O (which here varies based on the number of subjects S, the number of time points T, and the sampling rate R). We additionally included a random intercept for the different parcellations in this model. In the second part, we modeled the effects on model stasis of four factors: 1. the number of observations O, 2. the inverse of the number of free parameters per state DF (which varies based on the number of parcels N from each parcellation), 3. their interaction, and 4. FC similarity. In this SEM, we included both a random intercept and random slope of the effect of FC similarity for each parcellation. The number of observations O has again both a direct and an indirect (via FC similarity) effect on model stasis. An overview of all variables in both the real data and the simulated data can be found in Table 1.

**Table 1:** Overview over variables manipulated in real (HCP) data and simulations.

Variables	In real (HCP) data	In simulations
Between-subject variability $\delta_{bs}$	–	[0.1 … 1]
Within-session variability $\delta_{ws}$	–	[0.1 … 1]
Number of subjects S	50, 100, 200	20, 100
Number of timepoints T	100, 500, 1200	1200
Sampling rate R	1, $\frac{1}{2}$, $\frac{1}{3}$	1
Parcellation	GroupICA50, GroupICA100, PROFUMO50, DK80, Yeo100	GroupICA50
Number of parcels N	10, 25, 50, all parcels	10, 50 (all parcels)
Number of HMM states K	12, ({4,..60})	6, ({4,…60})
(Number of observations O)	O=S*T*R	
(Number of free parameters per state DF)	$DF=\frac{K*\left(K-1\right)+\left(K-1\right)+\frac{K*N*(N+1)}{2}}{K}$	
Outcome measures		
FC similarity	Time-averaged FC group correlation between all subjects	
Model stasis	Mean maximum fractional occupancy	

We used the piecewiseSEM-package (Lefcheck, 2016) in R (R Core Team, 2020) to fit the SEM models as a combination of linear and linear mixed effects models.

## 3 Results

We address the factors from the two hypotheses (3.1 Data hypothesis, 3.2 Estimation hypothesis) one by one, distinguishing between results from simulated data and real data (HCP data). Statistics from the full structural equation models (SEM) are summarised under Section 3.3.

### 3.1 Data hypothesis

We first investigated which aspects of the data influence the ability of a time-varying FC model to detect temporal changes in FC (data hypothesis). Namely, we tested the effects of between-subject variability and of within-session variability on FC similarity and on model stasis in simulated time series (Section 3.1.1). We then focussed on the effect of the parcellation used to extract time series from the HCP resting state data on FC similarity, on model stasis, and on the relationship between them (Section 3.1.2).

#### 3.1.1 Between-subject and within-session variability in simulated time series affect model stasis

Here, we show on synthetic data that large differences between subjects or small differences over time can cause the time-varying FC model to become static.

**Fig. 2:**
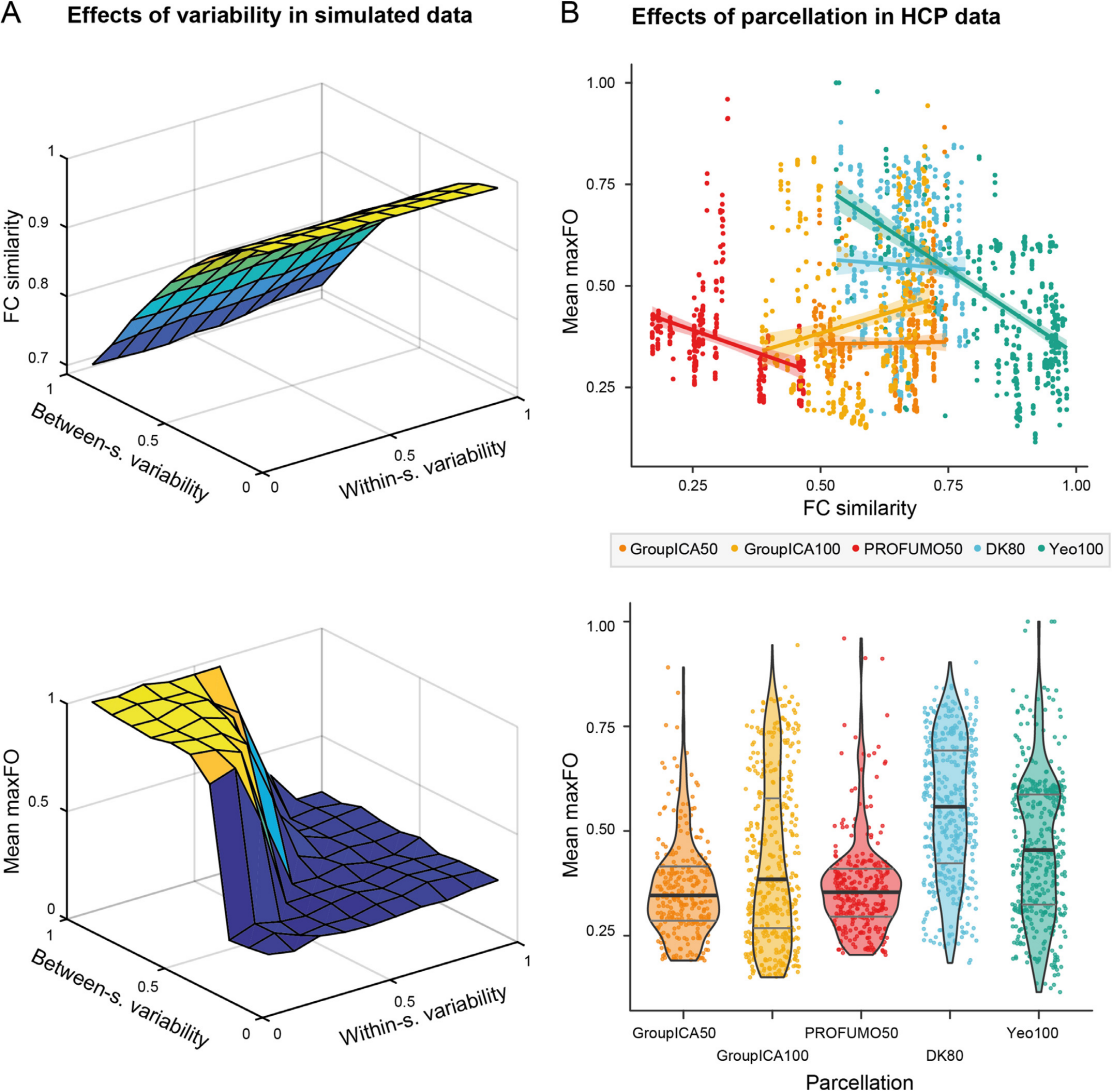
Evidence for the data hypothesis. (A) In the simulated data, between-subject variability but not within-session variability affects FC similarity between subjects (top panel). The bottom panel shows how between-subject and within-session variability affect model stasis (as measured by mean maxFO) in a time-varying FC model. In the graph area where between-subject variability is high and within-session variability is low, the model is static (yellow area). (B) In the real data, FC similarity and model stasis depend on the parcellation. We here represent each parcellation with a different color. In the top panel, we illustrate the linear regression line and corresponding 95% confidence interval between FC similarity and model stasis (represented by the mean maxFO statistic) within each of the parcellations. The graph shows how both the position and the slopes for these regression lines are different between parcellations. In the bottom panel, we show the distribution of mean maxFO values within each of the parcellations. The thick black line within each violin plot indicates the mean value of mean maxFO in the respective parcellation and the grey lines indicate their interquartile range. Dots within each parcellation correspond to runs with different dimensionality parameters as described under Section 2.1, i.e. different numbers of subjects S, time points T, sampling rates R, and (subsets of) parcels N. Note that the points in the bottom panel plot are jittered along the x-axis to better visualise all models (For interpretation of the references to color in this figure legend, the reader is referred to the web version of this article.).

In order to address the question of variability in the data, we simulated new data with different degrees of between-subject and within-session variability (described under Section 2.1.3). First, we calculated FC similarity of these new FC matrices, which confirmed that this measure robustly reflects between-subject variability $\delta_{bs}$, independently of within-session variability $\delta_{ws}$ (see Fig. 2A, top panel). In the full structural equation model (SEM), FC similarity (which we can measure in the real data) is near perfectly explained (standardised coefficient of −0.97, *p* < 0.0001) by between-subject variability $\delta_{bs}$ (the ground-truth in the simulations, which we cannot directly measure in the real data). We can therefore assume that, in the real data, FC similarity is a reliable proxy for between-subject variability.

FC similarity was not significantly affected by the number of observations O (i.e. by varying the number of subjects S) (coefficient: −0.02, *p* = 0.99). As hypothesised, model stasis depends on both between-subject and within-session variability, where high between-subject and low within-session variability cause the model to become static. Decreasing differences between subjects and increasing temporal variability in the data lead to a lower rate of model stasis. This is shown for an exemplary solution in Fig. 2A, bottom panel. In the full model, the effects of between-subject and within-session variability are of a similar magnitude, with standardised coefficients of −0.53 (*p* < 0.0001) for FC similarity and −0.54 (*p* < 0.0001) for within-session variability.

In summary, this indicates that the between-subject vs. within-session variability balance is an important contributor to model stasis. That is, if subjects in the dataset are very dissimilar, differences across time points need to be large in order for a time-varying FC model to be able to identify dynamically changing states. In real datasets, it may therefore be important to work towards high similarity between subjects while retaining temporal variation as much as possible during preprocessing. One central factor in achieving this may be the choice of parcellation, which we tested next .

#### 3.1.2 The parcellation affects FC similarity, model stasis, and the relationship between them

We next investigated the effect of the parcellation on FC similarity, on model stasis, and on the relationship between them. As we will see, FC similarity does not simply explain model stasis, but the choice of parcellation can strongly affect FC similarity, model stasis, and the relationship between these two variables.

Time courses were extracted from the HCP data in five different parcellations: We used three data-driven functional parcellations (GroupICA50, GroupICA100, and PROFUMO50, Beckmann et al., 2009; Harrison et al., 2015), one *a priori* defined functional parcellation (Yeo100, Schaefer et al., 2018), and one anatomical parcellation (DK80, Deco et al., 2021; Desikan et al., 2006). As shown in Fig. 2B, the choice of parcellation affects FC similarity, model stasis (as measured by mean maxFO), and the relationship between them. We included the parcellation as random intercept in the first part of the full SEM (predicting FC similarity) and as random intercept and slope in the second part of the full SEM (predicting model stasis). This increased the variance explained by the full SEM as compared to a model excluding the effect of parcellation by 32% (*R^2^_reduced_* = 0.40, *R^2^_full_* = 0.72). In the full SEM, the remaining effect of FC similarity on model stasis, i.e. the fixed effect not depending on parcellation, is not significant (coefficient −0.06, *p* = 0.80). This indicates that the effect of FC similarity on model stasis is not as straightforward as we hypothesised, but strongly depends on the parcellation. As opposed to our hypothesis that higher similarity between subjects in time-averaged FC decreases model stasis, the parcellations that, on average, created the most similar time-averaged FC matrices between subjects, increased model stasis the most.

Besides the parcellation, FC similarity is also significantly explained by the number of observations O, yielding a coefficient of 0.23 (*p* < 0.0001). Across all runs, the parcellations ranked from least to most model stasis are: 1. GroupICA50 (M: 0.36 ± 0.12 S.D.), 2. PROFUMO50 (M: 0.37 ± 0.12 S.D.), 3. GroupICA100 (M: 0.41 ± 0.20 S.D.), 4. Yeo100 (M: 0.46 ± 0.17 S.D.), 5. DK80 (M: 0.55 ± 0.16 S.D.). On average, the three data-driven functional parcellations used here outperformed both the example of an *a priori* functional and the example of an anatomical parcellation, in the model's ability to detect dynamic changes in FC.

### 3.2 Estimation hypothesis

Estimating a large number of free parameters per state from limited data poses a statistical challenge in the estimation of any model. We next quantified the influence of the number of free parameters per state and the number of observations on model stasis. We show that a high number of free parameters per state and a low number of observations can cause the model to become static. Additionally, we here show the effect of varying the number of states on model stasis.

#### 3.2.1 Varying the number of free parameters per state and the number of observations affect model stasis

**Fig. 3:**
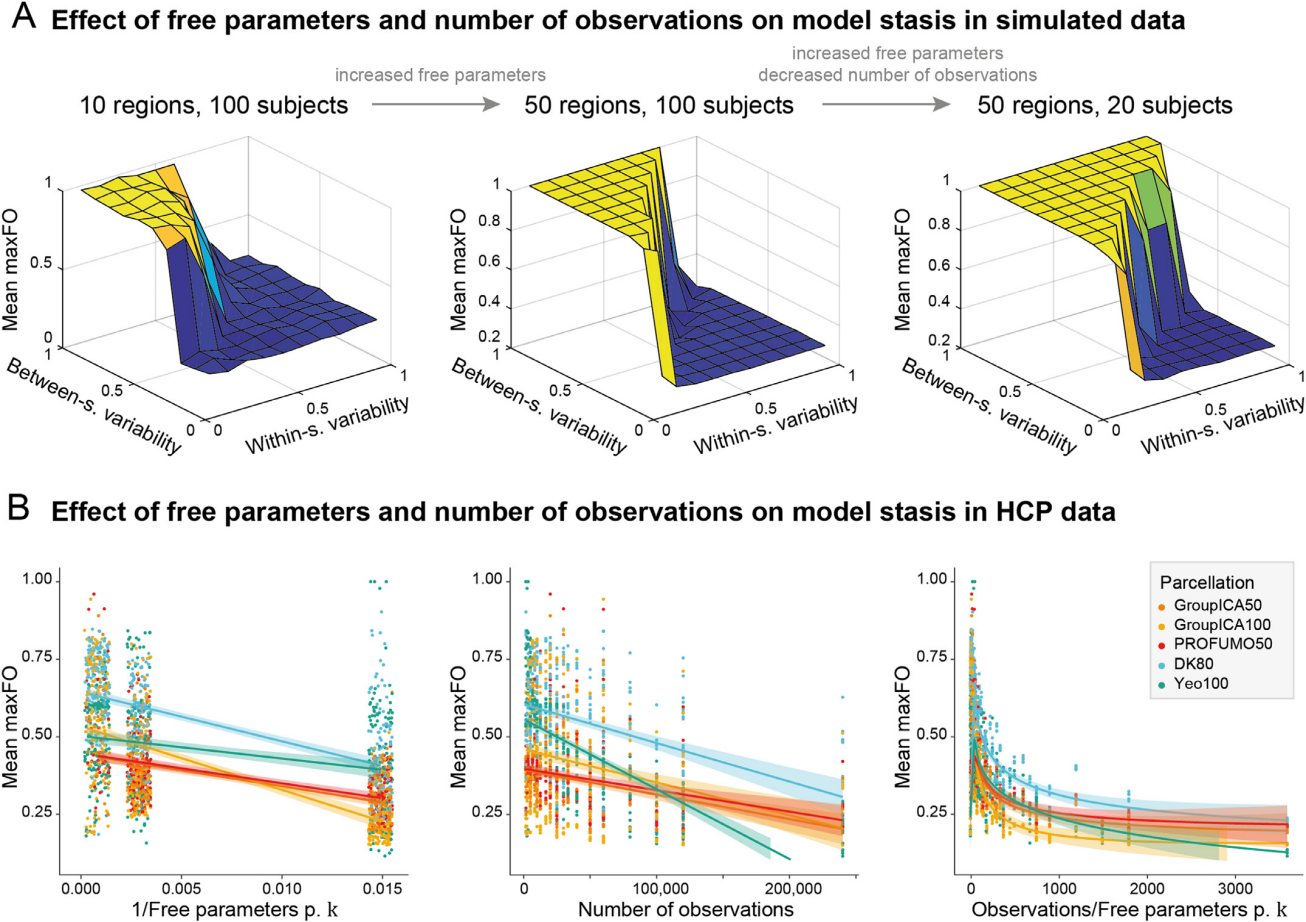
Evidence for the estimation hypothesis. (A) In the simulated data, we first increased the number of free parameters per state DF by manipulating the number of parcels N included from the parcellation (middle panel). This increased the yellow area of the graph, i.e. the area where the time-varying FC model is static. In addition to increasing the number of free parameters per state DF, we then also decreased the number of observations O by simulating fewer subjects S (right panel). This further increased the area where the time-varying FC model is static, so that it is now only possible for the model to detect dynamics (blue area) when between-subject variability is very low and within-session variability is very high. (B) In the real data, both decreasing the number of free parameters per state DF (left panel, where we show the inverse of the number of free parameters per state) and increasing the number of observations O (middle panel) reduce model stasis, as indicated by lower values in mean maxFO. Finally, the ratio of observations to free parameters per state (right panel) is a strong negative indicator of model stasis. This ratio is small in all models that are mostly static (high values of mean maxFO) and high in all models that are mostly dynamic (low values of mean maxFO). Given the differences between parcellations established in Section 3.1.2, we distinguish between parcellations in these plots. This distinction is here only for illustrative purposes and not included as random effects in the full SEM. The shaded area around each regression line represents the 95% confidence interval. Note that the points in the left panel plot are jittered along the x-axis to better visualise each model (For interpretation of the references to color in this figure legend, the reader is referred to the web version of this article.).

In the simulated data, we found that both increasing the number of free parameters per state DF by including more parcels of the parcellation (i.e. increasing N) and decreasing the number of observations O by simulating fewer subjects increase model stasis. This is illustrated in Fig. 3A where, compared to the models described above under Section 3.1.1 (plotted in the left panel), we increased the number of free parameters per state DF (middle panel), and additionally decreased the number of observations O (right panel). The area where the model becomes static (i.e. where mean maxFO is high, here shown in yellow) increases for both steps. In the full model, the standardised coefficients for the inverse of the number of free parameters per state DF is −0.16 (*p* = 0.0001) and for the number of observations O is −0.21 (*p* < 0.0001).

In the real data, the number of free parameters per state DF was manipulated by changing the number of parcels N as described under Section 2.1.2. As illustrated in Fig. 3B, both decreasing the number of free parameters per state DF (left panel) and increasing the number of observations O (middle panel) decreased model stasis in the HCP data. A low ratio of number of observations O to free parameters per state DF (right panel) is a strong indicator of model stasis. Based on the finding that model stasis strongly depends on the parcellation, we here also plot these effects for each parcellation separately. Please note that we plot the inverse of the number of free parameters per state in the left panel, so that the values in the right panel are the product of the two previous plots. In the full SEM, the coefficient of the inverse of the number of free parameters per state DF is −0.50 (*p* < 0.0001), the coefficient of the number of observations O is −0.30 (*p* < 0.0001) and the coefficient of their interaction is −0.06 (*p* = 0.02). As shown in the Supplementary Figs. 1 and 2, reducing the number of free parameters per state DF using the PCA- and HMM-PCA approaches similarly decreased model stasis. This effect was parcellation-dependent.

In a dataset with few observations, reducing the number of free parameters per state may therefore be vital for a time-varying FC model to detect dynamic changes in FC. If more data is available, it is possible to increase the number of free parameters per state and thus add detail to the model, e.g. by using a more fine-grained parcellation.

#### 3.2.2 Varying the number of states affects model stasis

**Fig. 4:**
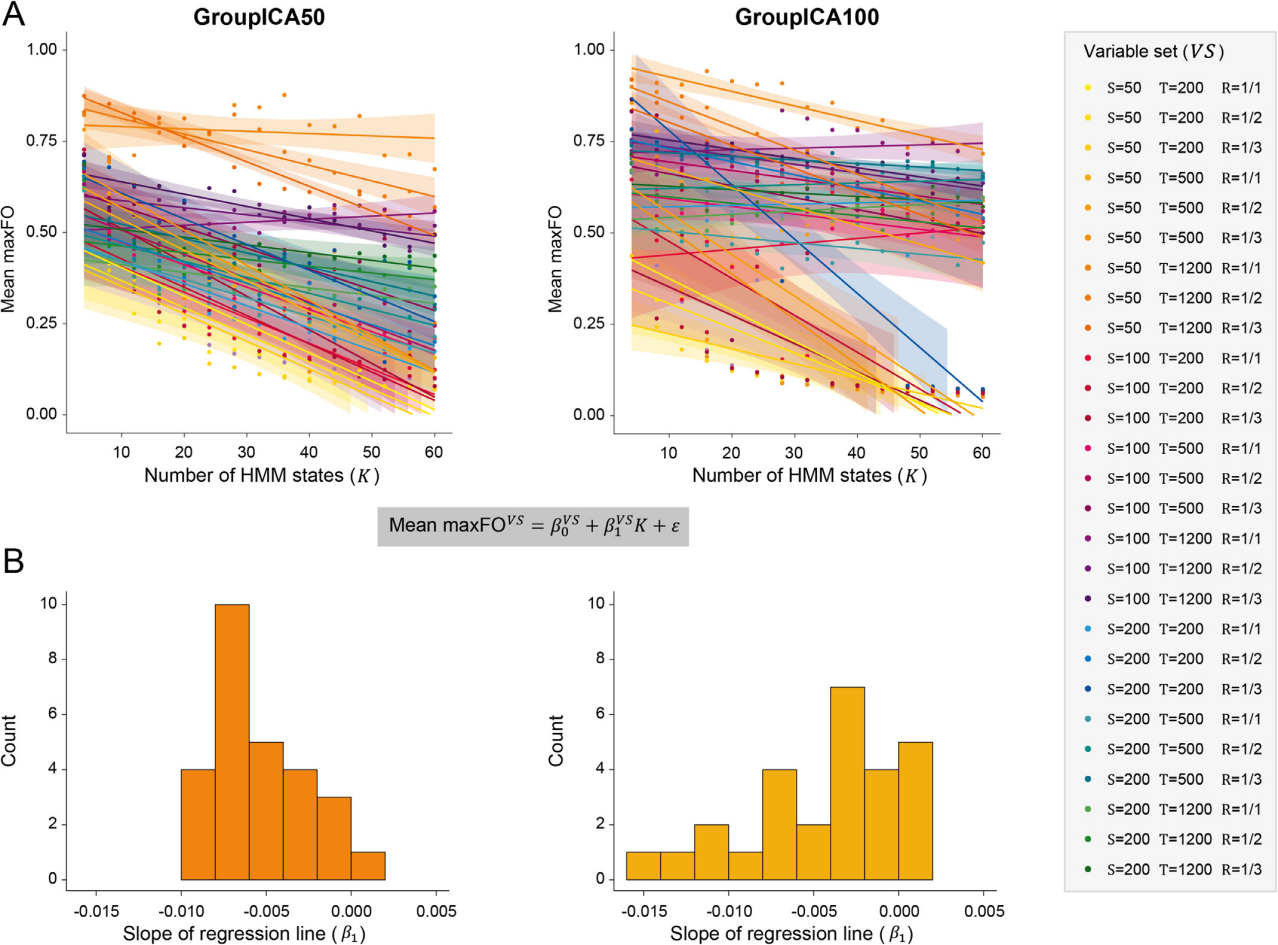
The number of states K in the model affects model stasis. (A) The number of HMM states K∈R{4,...60} (increasing in steps of 4) are shown on the x-axis. The individual model stasis outcomes of each individual model (as measured by mean maxFO) are shown on the y-axis. For each variable set VS, we also show the regression lines, as defined by the regression $Mean\;maxFO^{VS}=\;\beta_{0}^{VS}+\beta_{1}^{VS}K+\varepsilon$. Each color in the plots represents a different variable set VS. The shaded area around each regression line represents the 95% confidence interval. In the GroupICA50 parcellation (left panel), increasing the number of states K decreases model stasis in almost all variable sets VS, while in the GroupICA100 parcellation (right panel), increasing the number of states K decreases model stasis only in some variable sets VS, but does not affect it in others. (B) The histograms of slopes ($\beta_{1}$) of the regression lines in (A) shows a peak between −0.01 and −0.005 for the GroupICA50 parcellation (left panel) and shows several peaks, one below −0.01, one between −0.01 and −0.005, and a large portion around 0 for the GroupICA100 parcellation (right panel).

Focusing on the real data and the data-driven parcellations, we additionally tested how changing the number of states K in the model affects model stasis. Fig. 4. shows how, for a given variable set VS, increasing the number of states K either decreases model stasis or does not affect it. In Fig. 4A, the individual model stasis outcomes, as measured by mean maxFO, and regression slopes for K∈R{4,...60} are shown for all variable sets VS that were used to vary the number of observations O Fig. 4.B shows the histogram of the individual slopes of these regression lines, i.e., $\beta_{1}$ for all VS. In the GroupICA50 parcellation (left panel), increasing the number of states K decreases model stasis in almost all variable sets. The histogram of slopes $\beta_{1}$ in this parcellation peaks between −0.005 and −0.01. In the GroupICA100 parcellation (right panel), increasing the number of states K decreases model stasis in some variable sets and does not affect it in other variable sets. Again, the histogram of slopes $\beta_{1}$ has a large portion below 0. In summary, having more states has a tendency to reduce model stasis, as the estimation becomes more fractionated.

### 3.3 Synthesis of results

**Fig. 5:**
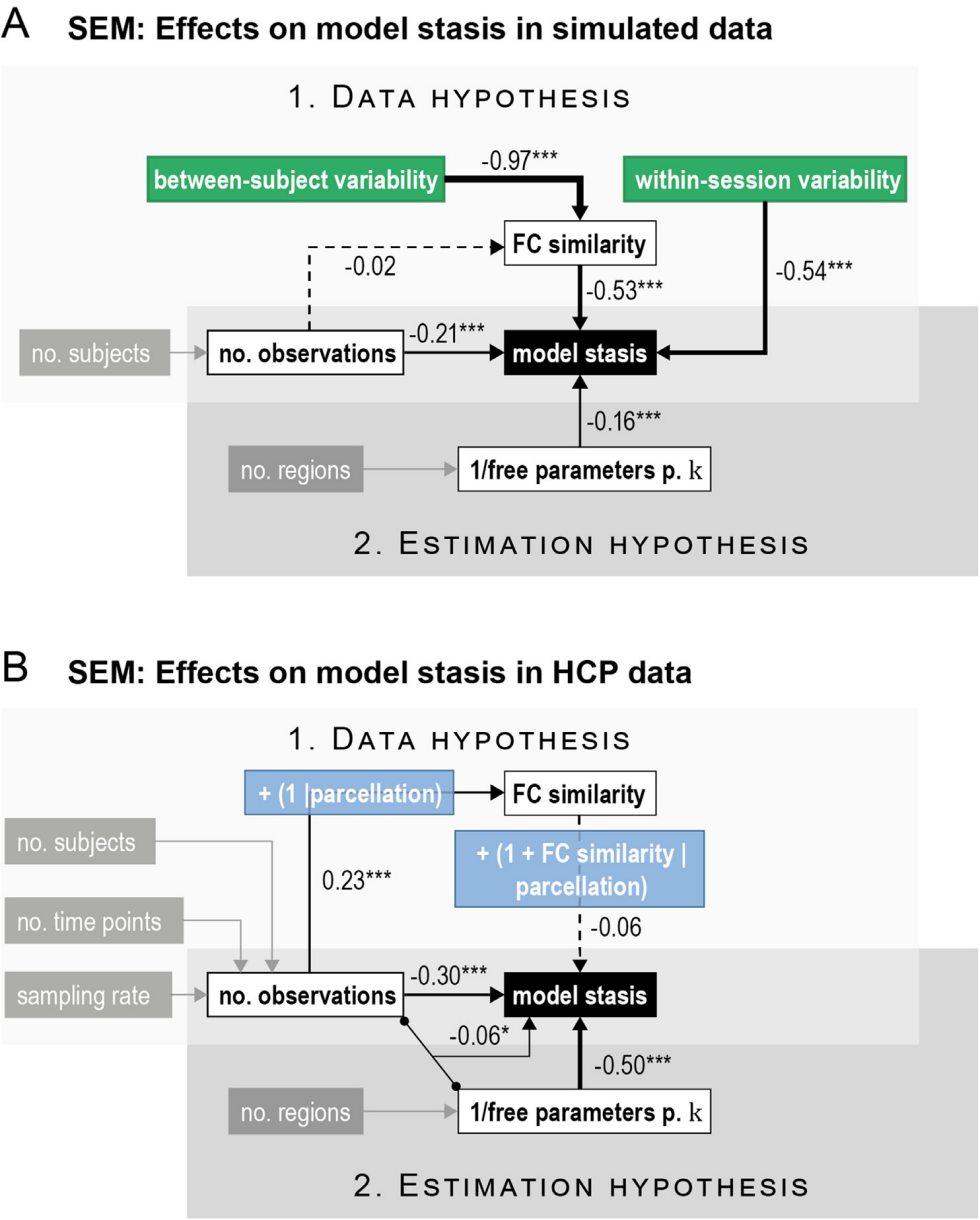
Full structural equation models (SEM). (A) On simulated data, FC similarity (which is almost perfectly explained by between-subject variability) and within-session variability strongly affect model stasis, providing compelling evidence for the data hypothesis. Coefficients corresponding to the estimation hypothesis are smaller, but still both the number of free parameters per state and the number of observations significantly affect model stasis. (B) On real data, the effect of the data hypothesis is less strong, as indicated by the smaller coefficients between FC similarity and model stasis. As explained in Section 3.1.2, variance in model stasis from the data hypothesis can be explained better by distinguishing between different parcellations. The number of free parameters per state and the number of observations, as well as their interaction strongly affect model stasis. Here, grey boxes indicate variables that are not explicitly modeled in the SEM, but which are constituting parts of another variable. White boxes represent predictor variables. Green boxes are synthetically manipulated variables in the simulated data. Blue boxes specify random effects that affect the underlying link. The black box indicates the main outcome variable. Arrow thickness is scaled to the corresponding coefficient strength. Significance levels are indicated by asterisks: ***p < 0.001, **p < 0.01, *p < 0.05 (For interpretation of the references to color in this figure legend, the reader is referred to the web version of this article.).

In order to compare the directed effect of all variables on model stasis, we finally modeled the influence of all factors described under Sections 3.1 and 3.2 using SEMs. We estimated separate models with a similar structure for the simulated and the real data, as described in Section 2.4. The structure and results of the SEM are summarised in Fig. 5. The first part of each model uses FC similarity as the outcome measure, while the second part uses model stasis as the outcome. This structure allows differentiating for instance between a direct effect of the number of observations on model stasis and an indirect effect of the number of observations on model stasis via FC similarity.

The full models explain 95% and 86% variance in FC similarity, and 68% and 72% in model stasis, respectively, for simulated and real data. Comparing the two hypotheses, in the simulated data we found more evidence for the data hypothesis than for the estimation hypothesis. In the real data, however, the evidence supporting the estimation hypothesis dominates the data hypothesis, particularly the number of free parameters per state. An apparent difference between the simulated data and the real data is the effect of FC similarity (standardised coefficients of −0.53*** in simulated data and of −0.06 N.S. in real data). It is also important to note that we use only one parcellation in the simulations, but five different parcellations in the real data. We show above that, in the real data, 32% of variance in model stasis is explained by the random effects of parcellations. This indicates that, rather than using overall FC similarity as a single indicator of model stasis, it is important to distinguish between different parcellations. Another important difference between simulated and real data is that the amount of between-subject and within-session variability can only be directly manipulated on the synthetic data. However, between-subject and within-session variability often differ to a large extent between real datasets and are therefore an important consideration when applying time-varying FC models.

Overall, we found evidence for all hypothesised effects. At the level of the data hypothesis, we showed in the simulations that low between-subject and high within-session variability reduce model stasis. Additionally, on real data, we showed that the choice of parcellation strongly affects time-averaged FC, model stasis, and the relationship between them. At the level of the estimation hypothesis, we presented evidence that a larger number of observations and fewer free parameters per state reduce model stasis, both on simulated and on real data.

## 4 Discussion

The ability of a time-varying FC model to identify temporally changing states on fMRI data depends on numerous factors, and can be attributed both to aspects of the data and to aspects of the model. Our findings indicate that model stasis is affected by the actual variability in the data, the parcellation used to extract time courses, and the ratio of the number of available observations to the number of free parameters per state in the model. To summarise when these models can be satisfactorily applied, we have compiled a short list of practical recommendations in Conclusions.

We first showed that large differences between subjects and/or small within-session FC modulations can cause a time-varying FC model to become static. This can be explained by the data-driven, unsupervised nature of the model, which aims at describing the most salient features of a dataset without imposing specific constraints about the recurrence of states across subjects. We also showed that FC similarity, model stasis, and the relationship between them are affected by the parcellation. In the example parcellations we used here, the three data-driven parcellations on average resulted in lower model stasis (i.e. they were found to be better models from the specific point of view considered in this paper) than the examples of *a priori* functional or anatomical parcellations. Although these conclusions might not necessarily generalise to all functional or anatomical parcellations, the effect was clear in this case. Understanding the reason behind these differences between parcellations is not straightforward, as there are several factors involved —such as differences in spatial distribution, cluster size, weighted vs. binary parcels, time course extraction, etc.— that may contribute to these differences, and have not been explicitly tested here. For instance, assuming the presence of “true” functional clusters in the data, data-driven functional parcellations are more likely to detect these clusters as parcels, resulting in a more efficient estimation of the temporal variance of these clusters. In theory, anatomical and *a priori* functional parcellations may, for example, split “true” functional clusters into several parcels, which could affect the balance between-subject and within-session variability in an artefactual manner. This might also explain our finding that, although the *a priori* functional and the anatomical parcellation create more similar time-averaged FC matrices between subjects, model stasis is higher in these parcellations on average.

Second, we showed that a high number of free parameters per state in the model can cause the model to become static, especially if too few observations are available to estimate these parameters. Here we showed that the model may become static when too many free parameters per state need to be estimated. This is because, if the data available for the estimation of time-varying FC is insufficient, avoiding all state switches might be the most parsimonious solution in terms of the model inference. This implies that the estimation of time-varying FC is a trade-off between the level of detail in the spatial domain and the accuracy of the temporal estimation.

We also showed that increasing the number of states in a model can decrease model stasis outcomes in some models. This result should be interpreted with caution, since, for example, a higher number of states also increases the chances of “random” switches at any timepoint, simply because more states are available; or could make the estimation of the state parameters less precise (because less data is available per state) therefore making it harder for a single state to successfully explain an entire subject's data. While high values in our model stasis outcome measure mean that the model failed in finding dynamics, low values do not necessarily imply that the model accurately detected meaningful dynamics, since this could just be due to having a noisier estimation.

Importantly, the factors we considered here are not exhaustive and therefore other variables related to overall data quality and model characteristics might also be relevant. In particular, a large dissonance between the model specification and the realities of the data could also be a reason why we could not detect time-varying FC. For instance, if temporal modulations in first-order statistics (the average pattern of activity within a state —i.e. the mean of the Gaussian distribution) were temporally independent from modulations in time-varying FC, this would violate the assumptions of the HMM and could potentially affect model stasis; in this case, modeling the mean as a separate temporal process would likely improve the estimation of time-varying FC (Pervaiz et al., 2022). Furthermore, we have shown in the supplementary results that our measure of model stasis must be regarded as a summary measure and that the distribution of FC can vary above and beyond that measure within the dataset. For example, model stasis does not necessarily affect all subjects or sessions in a dataset equally, so that the model might detect temporal changes in FC in certain subjects but not in others. In practice, it would be advisable to further inspect the actual state time courses of a model of interest across subjects and sessions.

It also remains to be seen how exactly model stasis may occur in other kinds of data or models. For instance, we have here only considered single-session fMRI data, which allowed investigating the effects of within-session and between-subject variability. Using multi-session fMRI recordings could address the interesting question of how within-subject (between-session) variability affects model stasis. Future work should also investigate these questions in EEG and MEG recordings, where FC is typically defined as a function of frequency. Another relevant next step would be considering different kinds of models (such as a mixture of Gaussian distributions, Bishop, 2006), although the logic of our conclusions is likely to remain valid—insofar as these models are based on covariance to assess FC (other definitions of FC exist that could bring in other factors to consider). One relevant difference between the HMM and other models is that the HMM assumes a Markovian transition probability matrix, which in practice regularises the state transitions and to some extent prevents noisy switching. Other models might have lower stasis as a consequence of having a higher estimation noise in that sense, but we do not expect this effect to be large. Other state-based models are also possible, like the Hidden semi-Markov model (HSMM) which is better able to characterise the duration of the state visits (Shappell et al., 2019). While these types of models may reduce potential spurious state transitions, they do not substantially differ in terms of their estimation of the measure we consider here, i.e. the proportion of the timecourse that a given state occupies. Regarding the simulation paradigm, our simulation model only generates data from Gaussian distributions, as assumed by the HMM; other biophysically more realistic simulations could also be informative (Erhardt et al., 2012). It should also be noted that we here only focussed on model stasis, because it is among the most fundamental measures of performance of a time-varying FC model. However, other evaluative measures, such as the ability to predict individual traits and behavior may be of interest when evaluating time-varying FC model performance, as shown in Pervaiz et al., 2020, Vidaurre, 2021, Pervaiz et al., 2022 and many other works. It is likely that some of the variables we here showed to reduce model stasis, such as higher similarity between subjects and fewer free parameters per state (as obtained, e.g., through a coarser parcellation), would indeed be disadvantageous when considering other evaluative measures or when conducting a time-averaged FC study. Finally, we were agnostic to the biological causes underlying between-subject and within-session differences, and chose to use only resting-state data. The potentially idiosyncratic way in which subjects perform a task, for instance, could be an important causal mechanism for these factors in task data, which might be explored in future work.

## 5 Conclusion

As we outlined in this article, the ability to estimate time-varying FC in fMRI data depends on several factors, which should be considered when planning and conducting a time-varying FC study. To avoid a time-varying FC model becoming static, we provide the following recommendations:

• Special care should be taken in reducing artefactual between-subject differences, e.g. by optimising registration and removing subject-specific artefacts, and in preserving meaningful temporal variance (i.e., non-artefactual) by refraining from preprocessing steps that average over time points like motion scrubbing or other more “aggressive” clean-up strategies. Less aggressive temporal preprocessing strategies, such as the ones recommended as part of the HCP resting state preprocessing guidelines (Smith et al., 2013b), that remove artefactual (e.g., motion-related or other physiological) temporal changes while preserving the signal's temporal variance are likely beneficial to avoid modeling dynamic changes due to motion rather than time-varying FC. Testing similarity in time-averaged FC between subjects may in some cases be useful as an indicator of problematic between-subject variability, but it can also be misleading in certain parcellations.
• The choice of parcellation used to extract time courses should be considered when planning a time-varying FC study. The data-driven functional parcellations we used here, such as Group ICA approaches, perform better than the examples we used for *a priori* functional or anatomical parcellations in detecting temporal changes in FC.
• The number of free parameters per state should ideally be not too large in relation to the number of observations, e.g. by using a parcellation with fewer parcels or components if necessary. Other options to reduce the number of free parameters per state include dimensionality reduction, e.g. using Principal Component Analysis (PCA), which however may affect the model in other ways (Vidaurre, 2021). Based on the HCP-dataset, we estimate as a rule of thumb that the ratio of observations to free parameters per state should not be inferior to 200.

In summary, meeting these requirements may help improving the robustness and reliability of time-varying FC methods and eventually increase replicability (Choe et al., 2017).

## CRediT authorship contribution statement

**C Ahrends:** Conceptualization, Methodology, Software, Formal analysis, Investigation, Writing – original draft, Visualization. **A Stevner:** Conceptualization, Methodology, Software, Writing – review & editing. **U Pervaiz:** Methodology, Data curation, Writing – review & editing. **ML Kringelbach:** Data curation, Supervision. **P Vuust:** Resources, Supervision, Funding acquisition. **MW Woolrich:** Methodology, Software, Writing – review & editing, Supervision. **D Vidaurre:** Conceptualization, Methodology, Software, Investigation, Writing – review & editing, Supervision, Funding acquisition.

## Declaration of Competing Interest

The authors declare that they have no known competing financial interests or personal relationships that could have appeared to influence the work reported in this paper.

## Appendix Supplementary materials

Image, application 1
