# Supplementary material for: Data and model considerations for estimating time-varying functional connectivity in fMRI
Source: Neuroimage. 2022 May 15;252:119026. doi: 10.1016/j.neuroimage.2022.119026 (PMC9361391; doi:10.1016/j.neuroimage.2022.119026)
Supplement: Supplementary file 1 [file mmc1.docx]

Supplementary Material: Data and model considerations for estimating time-varying functional connectivity in fMRI

Ahrends C, Stevner A, Pervaiz U, Kringelbach ML, Vuust P, Woolrich M and Vidaurre D


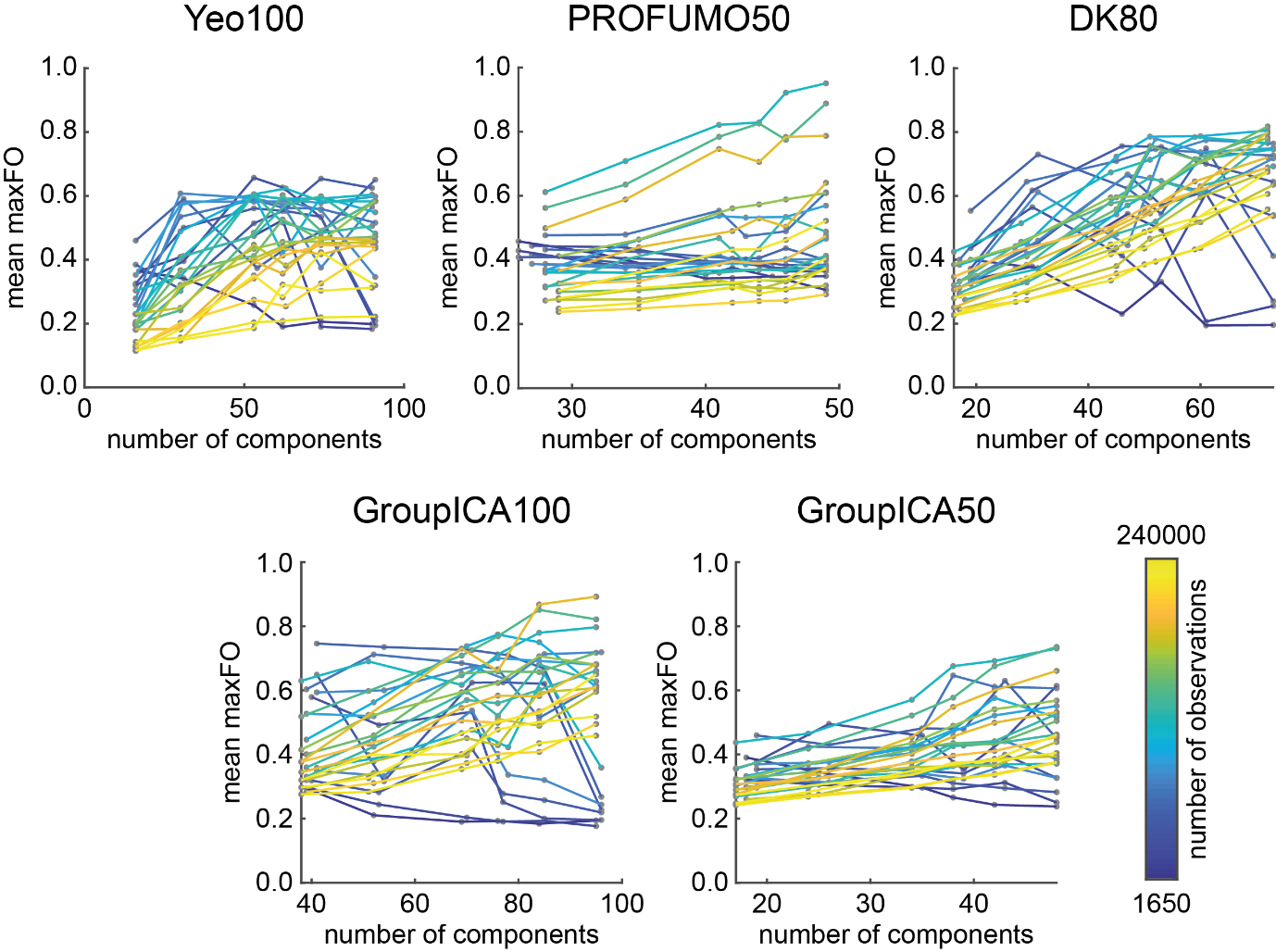


**Figure 1 Effects of PCA on model stasis by parcellation.** For each of the five parcellations, the plot shows how model stasis, as measured by mean maxFO, depends on the number of components to which the data were reduced through PCA. The successive points on each line graph explain 70, 80, 90, 93, 96, and 99% of the variance in their respective parcellation and number of observations. Overall, reducing the number of components, and thereby decreasing the number of free parameters, decreases model stasis. However, the effect differs between parcellations. Additionally, when the number of observations is very small (e.g. 1650 observations, corresponding to 50 subjects with 33 time points per subject), reducing the amount of variance through PCA may even reverse the effect such that fewer components increase model stasis.


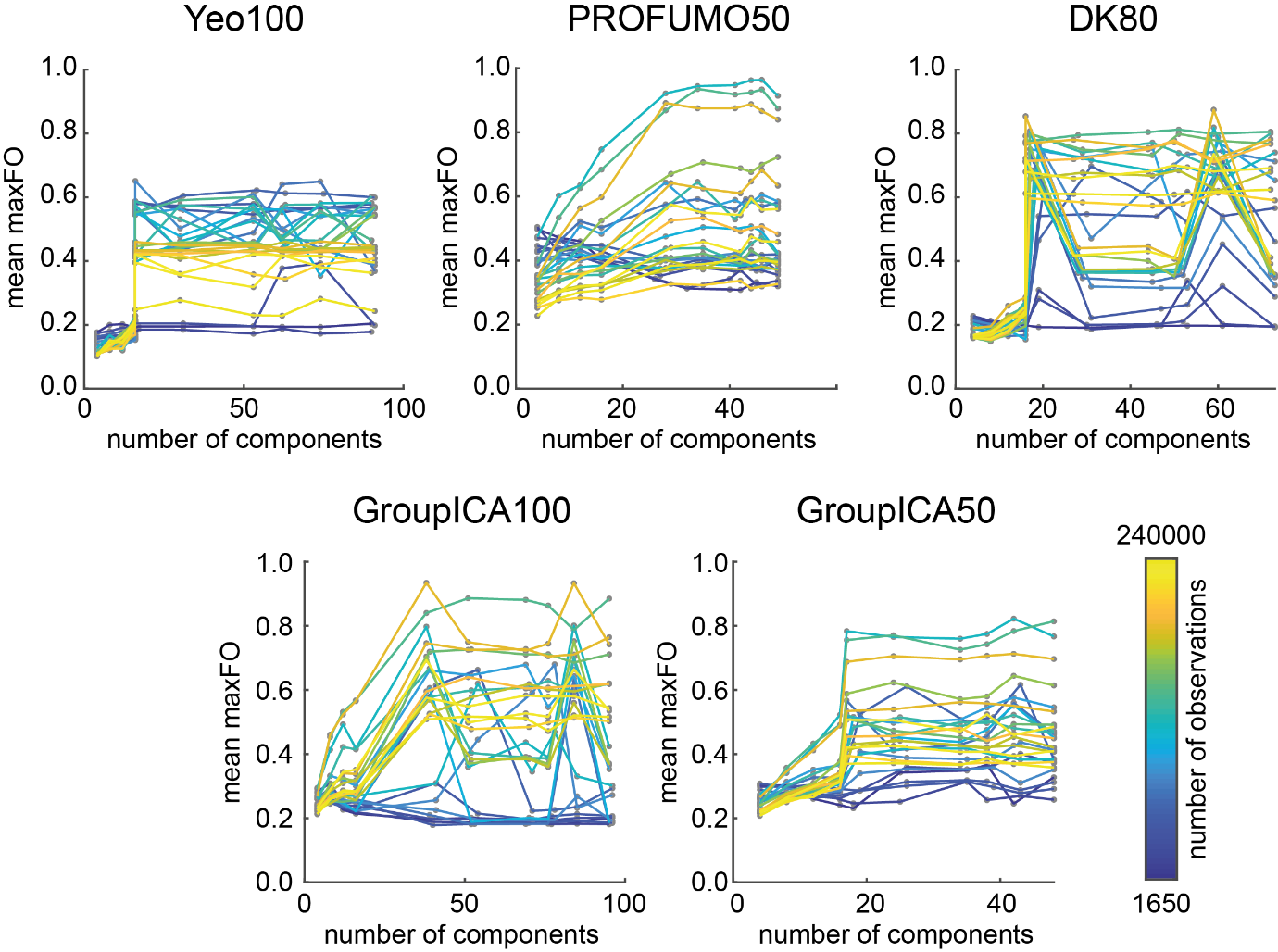


**Figure 2 Effects of HMM-PCA on model stasis by parcellation.** For each parcellation, the plot shows how model stasis, as measured by mean maxFO, depends on the number of components used to model each HMM-state as probabilistic PCA model. The first four points on each line graph correspond to 4, 8, 12, and 16 components. The remaining successive points on each line graph correspond to the number of components that explained 70, 80, 90, 93, 96, and 99% in the PCA-approach (i.e. in Supplementary Figure 1). In certain cases, reducing the number of components using the HMM-PCA approach decreases model stasis, e.g. when the number of components is very small and the number of observations is large. However, this effect does not persist for larger numbers of components, likely because this approach does not reduce the number of free parameters at the same degree as PCA does. Similar to PCA, when the number of observations is very small, fewer components may not affect or even increase model stasis. For details about the HMM-PCA approach, see Vidaurre, 2021.


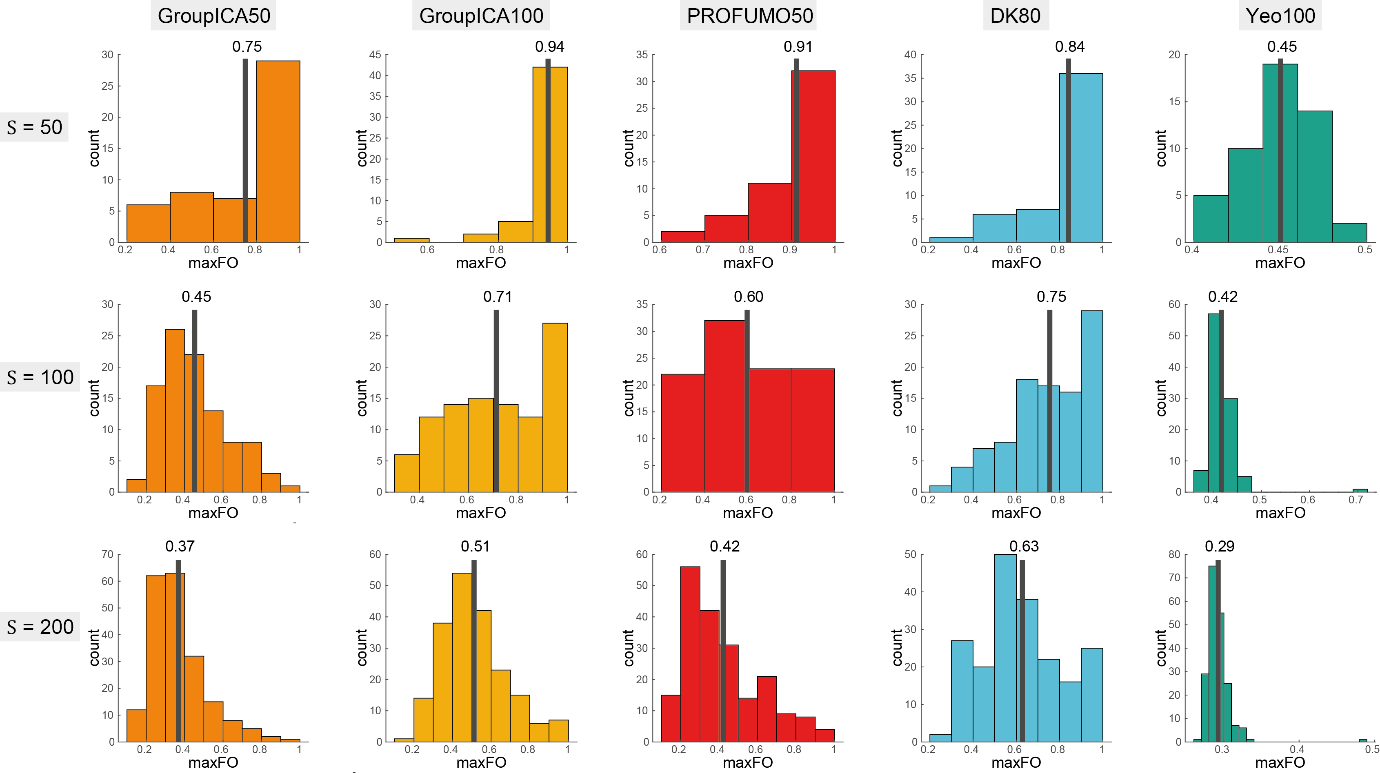


**Figure 3 Example distributions of maxFO for different configurations.** The histograms show the maxFO distributions across subjects for a given configuration of settings. We use the mean maxFO, indicated in each histogram as a thick vertical line with the corresponding value written above each line, to quantify model stasis. The columns are different parcellations and the rows are runs with different numbers of subjects. All example histograms depicted here are runs using the full parcellation (i.e., $N=50$ for GroupICA50 and PROFUMO50, $N=100$ for GroupICA100 and Yeo100, and $N=80$ for DK80), all timepoints (i.e.,, $T=1200$), and a sampling rate $R=1$. Please note that these are only examples of specific runs, such that it looks, e.g. as though runs in the Yeo100 parcellation have less model stasis, but this pattern does not persist when considering all combinations of variables, as we demonstrate in the Results.
